# The first comprehensive phylogenetic and biochemical analysis of NADH diphosphatases reveals that the enzyme from *Tuber melanosporum* is highly active towards NAD^+^

**DOI:** 10.1038/s41598-019-53138-w

**Published:** 2019-11-14

**Authors:** Antonio Ginés García-Saura, Rubén Zapata-Pérez, Ana Belén Martínez-Moñino, José Francisco Hidalgo, Asunción Morte, Manuela Pérez-Gilabert, Álvaro Sánchez-Ferrer

**Affiliations:** 1 Department of Biochemistry and Molecular Biology-A, Faculty of Biology, Regional Campus of International Excellence “Campus Mare Nostrum”, University of Murcia, Campus Espinardo, 30100 Murcia, Spain; 2 Lab. Genetic Metabolic Diseases F0-211, Academic Medical Center (AMC), 1105 AZ Amsterdam, The Netherlands; 3 Department of Plant Biology, Faculty of Biology, University of Murcia, Campus Espinardo, 30100 Murcia, Spain; 4 Murcia Biomedical Research Institute (IMIB-Arrixaca), 30120 Murcia, Spain

**Keywords:** Hydrolases, Biotechnology

## Abstract

Nudix (for nucleoside diphosphatases linked to other moieties, X) hydrolases are a diverse family of proteins capable of cleaving an enormous variety of substrates, ranging from nucleotide sugars to NAD^+^-capped RNAs. Although all the members of this superfamily share a common conserved catalytic motif, the Nudix box, their substrate specificity lies in specific sequence traits, which give rise to different subfamilies. Among them, NADH pyrophosphatases or diphosphatases (NADDs) are poorly studied and nothing is known about their distribution. To address this, we designed a Prosite-compatible pattern to identify new NADDs sequences. *In silico* scanning of the UniProtKB database showed that 3% of Nudix proteins were NADDs and displayed 21 different domain architectures, the canonical architecture (NUDIX-like_zf-NADH-PPase_NUDIX) being the most abundant (53%). Interestingly, NADD fungal sequences were prominent among eukaryotes, and were distributed over several Classes, including Pezizomycetes. Unexpectedly, in this last fungal Class, NADDs were found to be present from the most common recent ancestor to Tuberaceae, following a molecular phylogeny distribution similar to that previously described using two thousand single concatenated genes. Finally, when truffle-forming ectomycorrhizal *Tuber melanosporum* NADD was biochemically characterized, it showed the highest NAD^+^/NADH catalytic efficiency ratio ever described.

## Introduction

Nudix or NUDT (Nudix-type)^1^ hydrolases are Mg^2+^/Mn^2+^-dependent enzymes active towards nucleoside diphosphates linked to other moieties (X)^2^ and forming a superfamily distributed throughout the phylogenetic scale with more than 200,000 entries in the UniProtKB database. They share the characteristic conserved sequence required for substrate catalysis named the Nudix box (GX_5_EX_7_REUXEEXGU), where U represents a bulky hydrophobic amino acid, usually Ile, Leu or Val^2–5^. Nucleotide sugars, diadenosine polyphosphates, nucleoside triphosphates, ADP-ribose, NADH and NAD^+^ are among their most common substrates^6^. In addition, some Nudix hydrolases have the ability to degrade protein-conjugated ADP-ribose, as is the case with human NUDT16^7,8^. These compounds participate in crucial processes that require tight regulation. In fact, some Nudix hydrolases are overexpressed following cellular stress in order to recover homeostasis^9^.

These enzymes can be divided into subfamilies depending on specific sequence traits that are involved in substrate recognition, as first stated by Bessman´s group when they studied four ADP-ribose pyrophosphatases^3^. Thus, a conserved proline was found 16 amino acids downstream the Nudix box for all members of the ADP-ribose pyrophosphatase subfamily, whereas an invariant tyrosine designated another subfamily, the diadenosine polyphosphate pyrophosphatases^3^. Finally, an array of eight conserved amino acids (SQPWPFPXS) was seen to be characteristic of NADH pyrophosphatases or diphosphatases (NADDs)^3,10^. The first member of this last family was biochemically characterized in *Escherichia coli* as the protein product of the *nudC* gene, formerly known as *orf*257, which catalyses the hydrolysis of NAD(H) to AMP and NMN(H)^11^. Several other NADH pyrophosphatases have been described in different organisms, such as mouse NUDT13^12^, human NUDT12^9,13^, and *Saccharomyces cerevisiae* and *Caenorhabditis elegans* NPY1^14^. In addition, *Arabidopsis thaliana* present more than one NUDT representative, including NUDT1, NUDT2, NUDT6, NUDT7, NUDT10 and NUDT19^10,15–17^. However, AtNUDT1 activity was negligible at Mn^2+^ physiological levels^18^. AtNUDT2, 6, and 7 hydrolyse both ADP-ribose and NADH with almost equal catalytic efficiencies, whereas AtNUDT10 prefers ADP-ribose over NADH, and AtNUDT19, NADPH over NADH^10^. Of special interest are NADH pyrophosphatases from pathogenic organisms, such as those of *Mycobacterium* species (*M. tuberculosis, M. bovis* and *M. smegamatis*), which have been found to be involved in the degradation of the active forms of anti-tuberculosis drugs, such as isoniazid (INH-NAD) and ethionamide (ETH-NAD)^19,20^. In addition, NADH pyrophosphatase plays a crucial role in the assimilation of exogenous NAD^+^ in *Salmonella typhimurium*^21^, whereas in *Haemophilus influenzae*, it has also been described as the enzyme responsible for the growth of the pathogen in NAD^+^ containing media^22^. Interestingly, *E. coli* NudC has also been recently related with the RNA-decapping process, since it was able to efficiently remove the NAD^+^ cap in different prokaryotic RNAs, hydrolysing the pyrophosphate bond to produce nicotinamide mononucleotide (NMN) and 5′-monophosphate RNA^23,24^.

However, all the above studies have only provided a partial view of the NADH diphosphatase subfamily diversity. To address this, we have carried out an extensive bioinformatic analysis, taking into account other important amino acids recently described in the EcNudC structure^6,23^, giving rise to a new Prosite-compatible NADD pattern, which expands the previously published NADH diphosphatase sequence array. This *in silico* study provides the first phylogenetic distribution of NADDs, and also gives a picture of their domain architectures. The result revealed that NADH diphosphatases represent a small number of sequences (about 3%) of the Nudix superfamily. In addition, bacterial sequences are more abundant than those of eukaryotic organisms. However, among the latter, fungal sequences are profuse and widely distributed among different Classes, including Pezizomycetes, which produce relatively large fruiting bodies (apothecia) from epigeous, semi-hypogeous to hypogeous (truffles) origin^25^. Some of the last mentioned truffles are highly prized gastronomic delicacies, such as the black truffle of Périgord (*Tuber melanosporum* Vittad.)^26^. Of note, and totally unexpected, is the fact that the phylogenetic study made with known Pezizomycetes NADD sequences corroborated a previously reported molecular phylogeny carried out with several hundred conserved concatenated single-copy protein-coding genes^27^. In addition, the cloning and kinetic characterization of *T. melanosporum* NADD uncovers a new efficient biocatalyst with the highest NAD^+^/NADH catalytic efficiency ratio ever described.

## Results

### NADH pyrophosphatases represent a small but diverse group in the Nudix superfamily

**Figure 1:**
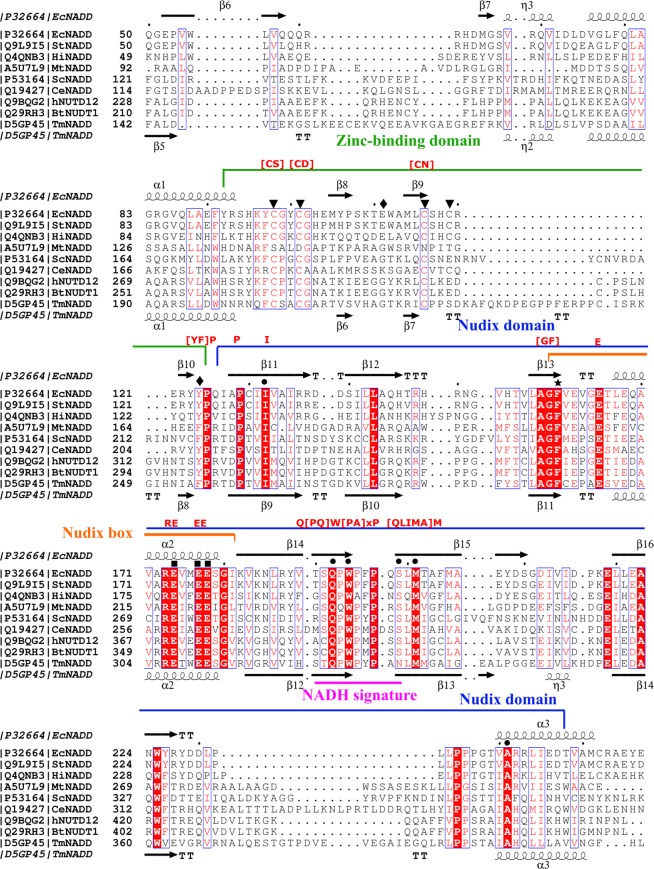
Multiple sequence alignment of NADH diphosphatases. The sequence of EcNudC (UniProtKB accession number, P32664) was aligned with its homologs from *Salmonella typhimurium* (StNADD, Q9L9I5), *Haemophilus influenza* (HiNADD, Q4QNB3), *Mycobacterium tuberculosis* (MtNADD, A5U7L9), *Caenorhabditis elegans* (CeNADD, Q19427), *Saccharomyces cerevisiae* (ScNADD, P53164), human (hNUDT12, Q9BQG2), *Bos taurus* (BtNUDT12, Q29RH3) and *Tuber melanosporum* (TmNADD, D5GP45). The four cysteine residues (C98, C101, C116 and C119) from the zinc-binding domain are marked with inverted triangles (▼). Residues involved in NAD^+^-binding pocket from the Nudix domain are marked with circles (●), whereas those from the zinc-binding domain with diamonds (♦). Catalytic residues are denoted with squares (■) and F160, involved in the adenine base binding via a π-π interaction, is marked with a star (⋆). The key amino acids of the Prosite pattern are shown in red letters above the alignment.

*E. coli* NudC (aka EcNADD) structures (PDB codes 5IW4, 5IW5 and 5ISY) (Supplementary Fig. S1A) and the sequence alignment with other biochemically characterized NADH pyrophosphatases (Fig. 1) were used to determine key sequence features to scan the UniProt database in an attempt to discover new canonical NADD members. The monomeric structure consists of an N-terminal domain (residues 1–92, EcNudC numbering) (aka NUDIX-like) and a C-terminal Nudix domain (residues 126–257, aka NUDIX, Fig. 1), formed by a six-stranded mixed sheet (β11–β16) sandwiched between two perpendicular α-helices (α2 and α3) (Fig. 1, Supplementary Fig. S1B). These two domains are separated by a zinc-binding domain (aka zf-NADH-PPase) (residues 93–125), which takes part in the dimerization of NudC and protrudes from the NTD^6,23^. The zinc is coordinated with four cysteine residues (C98, C101, C116 and C119) (Fig. 1, triangles, Supplementary Fig. S1B), of which, the first three are mainly conserved in described NADDs with the exception of that of *Mycobacterium tuberculosis* (Fig. 1). The NAD^+^ is bound into a pocket mainly found in the Nudix domain and with the participation of two amino acids from the zinc-binding domain, the non-conserved E111 and Y124 (Fig. 1, diamonds; Supplementary Fig. S1C). This last residue is involved in the adenine base binding via a π-π interaction with the aromatic ring of F160 (Fig. 1, star) from subunit A^23^. The nicotinamide moiety binds in a cavity comprising several bulky hydrophobic residues (I132, W194 and M201), in addition to Q192 and A241 (Fig. 1, circles), the last two residues being involved in the hydrogen bonding with the amide group of nicotinamide. These amino acids together with S199 (Fig. 1, circles), which interacts with the 3-hydroxyl group of the nicotinamide ribose, form the conserved eight-amino acid motif (SQPWPFPXS) downstream from the Nudix box (residues 159–181) (aka NADH signature) (Fig. 1), which has previously been used to classify the NADH hydrolase subfamily^3^. Based on the relevance of the above mentioned amino acids, the catalytic residues in the Nudix box (E174, E177 and E178) (Fig. 1, squares) and the essential role of NudC dimerization in substrate recognition, the Prosite-compatible pattern **[CS]x(2)[CD]x(12,15)[CN]x(5,35)[YF]**Px(3)Px(2)Ix(25,32)**GFx(4)Ex(7)REx(2)EE**x(13,14)**Q[PQ]W[PA]xP**x(2,9)[QLIMA]M was designed (Fig. 1, red letters; Supplementary Fig. S1C). This pattern covers the distance between C98 and M201, and basically represents the zinc domain (**[CS]x(2)[CD]x(12,15)[CN]x(5,35)[YF]**) and the conserved amino acids in the Nudix domain without β16 and α3 (Fig. 1), which includes the Nudix Box (**GFx(4)Ex(7)REx(2)EE**) and part of the conserved eight-amino acid motif, known as NADD signature (**Q[PQ]W[PA]xP**) (Supplementary Fig. S1D).

**Figure 2:**
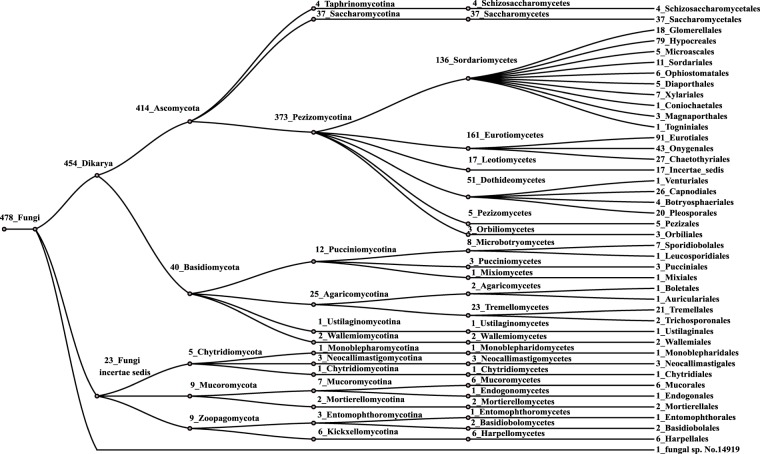
NADD sequence distribution in fungi. Scheme showing the abundance of NADD sequences in the different phylogenetic levels of the Fungi kingdom. The number represents the sequences found.

When this pattern was scanned against a Nudix protein sequence database composed of 234,112 sequences obtained using the Prosite pattern PS51462 (Nudix) in the UniprotKB database (including Swiss-Prot and TrEMBL; release 2018–08), 7479 sequences (3,2%) were NADDs. Among them, 90% were from Bacteria, 9.4% from Eukaryota, 0.24% from Archaea and the rest from metagenomic sources. No viral sequences were obtained. In Archaea, they were basically found in the Methanomicrobia Class, whereas in Bacteria, they are almost equally distributed between Gammaproteobacteria (39%), Alphaproteobacteria (30%) and Actinobacteria (23%), with two Orders distinguished by their great number of sequences, Enterobacterales (1008) and Rhizobiales (809). In Eukaryota, the sequences corresponding to mammals (80), birds (35) and bony fishes (26), together with those of roundworms (31), are particularly numerous, although the largest number belongs to Fungi (478). Among them, Ascomycota is the most abundant (86.6%), followed by Basidiomycota (8.3%), Chytridiomycota (1.0%), Mucoromycota (1.8%), Zoopagomycota (1.8%), and one sequence from the fungal sp. No.14919 (0.2%) (Fig. 2).

In addition, although NADDs are distributed among different fungal subphyla, Pezizomycotina (373) and Saccharomycotina (37) are clearly the most important in the retrieved sequences, followed by Agaricomycotina (25) (Fig. 2). Furthermore, within Pezizomycotina, the classes Eurotiomycetes (161) and Sordariomycetes (136) are relevant, since they include several sequences from *Fusarium* spp. or *Aspergillus* spp. NADDs are also represented in the Pezizomycotina basal groups, such as Orbiliomycetes and Pezizomycetes, with three and five sequences, respectively (Fig. 2). In particular, they were present in truffle-forming ectomycorrhizal species that are economically important gourmet delicacies, such as the aromatic Périgord black truffle (*Tuber melanosporum* Vittad.) (UniProtKB accession number: D5GP45), Burgundy truffle (*T. aestivum* Vittad.) (A0A292PI49), Piedmont white truffle (*T. magnatum* Pico) (A0A317SZB3) and whitish edible truffle (*T. borchii* Vittad.) (A0A2T6ZTD3).

To contextualise the results described above, the distribution of NADDs was also compared with the entire Nudix hydrolase family and another protein family, Peptidase S8 (Subtilisin and Subtilisin-like proteases) (Table 1). The results obtained showed that NADDs were less frequent in Archaea (0.24%) than in Nudix hydrolases (2.5%) and the Peptidase S8 family (4.2%). On the other hand, the percentage of NADDs in Fungi was almost double to that found in the entire Nudix family (6.4% vs. 3.6%), but lower to that found in the Peptidase S8 family (10.5%). The same percentage distribution pattern (0.07% vs 0.03%, 0.09%) was observed in the Pezizomycetes Class (Table 1). Moreover, although the NADD and NUDIX sequences are overrepresented in Bacteria, as they are in the UniProt TrEMBL database (~74%) compared to Eukaryota (~24%) (https://www.uniprot.org/uniprot/?query=reviewed:no#orgViewBy), when the latter kingdom was considered alone, NADDs appeared to be more represented in Fungi (68%) than in the NUDIX (39%) and Peptidase S8 (38%) families, respectively (Table 1). This higher presence was also found in the Ascomycota phylum (59% vs. 28%) and in the Pezizomycetes class (0.7% vs. 0.4%) (Table 1).

**Table 1:** Comparative distribution of sequences (%) in different protein families.

Taxonomic level	NADD	%	Nudix	%	Peptidase S8	%
Archaea	0.24		2.6		4.2	
Bacteria	90.1		86.4		67.2	
Eucaryota	9.4	100	9.4	100	27.3	100
Fungi	6.4	68.0	3.6	38.9	10.5	38.3
Ascomycota	5.5	58.9	2.6	27.9	7.9	28.8
Pezizomycetes	0.07	0.71	0.03	0.36	0.09	0.32

The sequences obtained using the Prosite pattern PS51462 (Nudix) in the UniprotKB database (including Swiss-Prot and TrEMBL; release 2018-08) for NADDs subfamily (7479), and sequences from NUDIX (234112) and Peptidase S8 (125470) families were retrieved using Retrieve/ID mapping tab from UniProt (https://www.uniprot.org/uploadlists/) and viewed using the corresponding Taxonomy link.

### NADH pyrophosphatases show multiple domain architectures apart from the canonical

**Figure 3:**
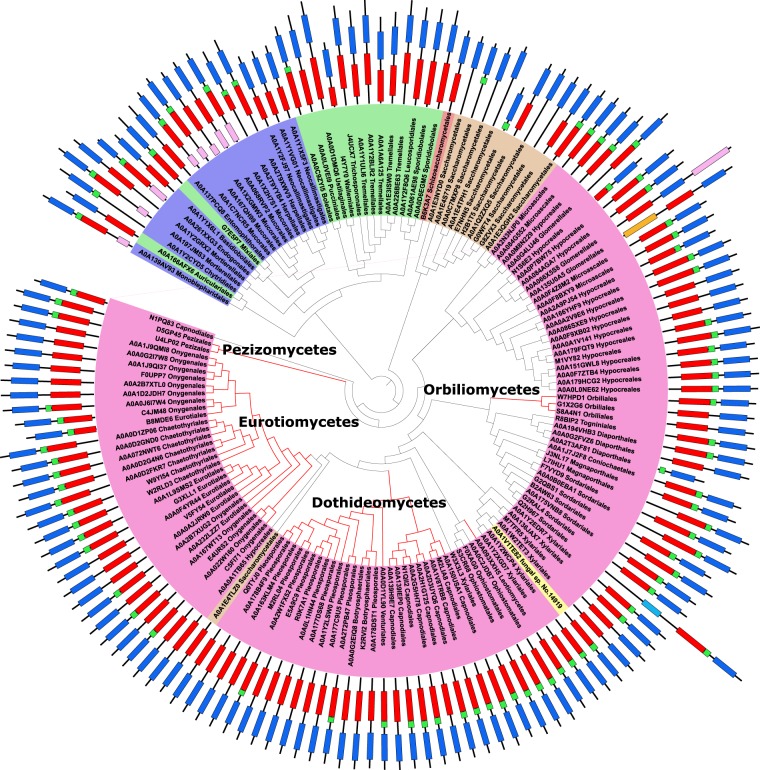
Phylogenetic analysis of fungal NADDs. The Neighbour-Joining (NJ) tree with 1000 replicates was constructed using MAFFT server. Protein domain architecture is shown beside each protein code: NUDIX-like (red), zf-NADH-PPase (green), NUDIX (blue) and ANK (purple). Fungal sequences are summarized in Supplementary Table S1.

All the above-mentioned truffle NADDs have the canonical NADD domain architecture (i.e. NUDIX-like_zf-NADH-PPase_NUDIX) (Fig. 3, Supplementary Table S1), which is also present in 54% of the fungal sequences (Table 2). The second most abundant NADD domain architecture in fungi is NUDIX-like_NUDIX (36%), followed by zf-NADH-PPase_NUDIX and NUDIX with 4% and 3%, respectively (Fig. 3, Table 2). In addition, there are other fungal minority domain architectures with a linear solenoid structure called ankyrin repeat domains (ANK) involved in protein-protein interactions, such as Ank5_NUDIX-like_zf-NADH-PPase_NUDIX (4), Ank2_NUDIX-like_NUDIX (2), Ank4_NUDIX-like_NUDIX (1) and Ank2_NUDIX-like_zf-NADH-PPase_NUDIX (2) (Fig. 3, Table 2). This last domain organization is similar to that described for human peroxisomal NUDT12^9^. In addition, there are also some unusual domain architectures, one with an ADP ribosylation factor (ARF) domain that function as a regulator of vesicular traffic, three with an actin remodelling motif (NUDIX-like_NUDIX_Arf), one isochorismatase_NUDIX-like_NUDIX and one MULE_NUDIX-like_zf-NADH-PPase_NUDIX (Table 2). The last MULE domain is an all-beta structure that is found in Mutator-like elements (MULE)-encoded transposases, which are related with DNA transposable sequences that can move from one locus to another in the genome^28,29^.

**Table 2:** Domain architecture distribution (%) in NADD and Nudix hydrolase families.

Domain architecture	Fungal NADDs	NADDs	NUDIX
NUDIX-like_zf-NADH-PPase_NUDIX	54.0	53.5	2.5
zf-NADH-PPase_NUDIX	4.0	36.8	1.4
NUDIX-like_NUDIX	36.0	4.5	0.61
NUDIX	3.1	2.8	83.4
Ank-2_NUDIX-like_zf-NADH-PPase_NUDIX	0.42	1.8	0.099
Ank-5_NUDIX-like_zf-NADH-PPase_NUDIX	0.84	0.29	0.02
Ank-2_NUDIX-like_NUDIX	0.42	0.08	0.006
NUDIX-like_NUDIX_Arf	0.63	0.04	0.003
Abhydrolase-6_NUDIX-like_zf-NADH-PPase_NUDIX	N.D.	0.027	0.002
Abhydrolase-6_zf-NADH-PPase_NUDIX	N.D.	0.027	0.002
Ank-2_zf-NADH-PPase_NUDIX	N.D.	0.027	0.002
Ion-trans-2_TrkA-N_NUDIX-like_zf-NADH-PPase_NUDIX	N.D.	0.027	0.002
01#Aa-trans_02#Aa-trans_NUDIX	N.D.	0.013	0.001
Ank-4_NUDIX-like_NUDIX	0.21	0.013	0.001
Ank-5_NUDIX-like_zf-NADH-PPase_NUDIX_Prefoldin	N.D.	0.013	0.001
Ank-5_zf-NADH-PPase_NUDIX	N.D.	0.013	0.001
DUF2805_zf-NADH-PPase_NUDIX	N.D.	0.013	0.001
HIT_NUDIX-like_zf-NADH-PPase_NUDIX	N.D.	0.013	0.001
Isochorismatase_NUDIX-like_NUDIX	0.21	0.013	0.001
MULE_NUDIX-like_zf-NADH-PPase_NUDIX	0.21	0.013	0.001
NUDIX-like_OrfB-Zn_ribbon_NUDIX	N.D.	0.013	0.001

The domain architectures of the sequences obtained using the Prosite pattern PS51462 (Nudix) in the UniprotKB database (including Swiss-Prot and TrEMBL; release 2018-08) for fungal NADDs (478). NADDs (7479) and NUDIX (234112) sequences were retrieved from the Pfam web site (https://pfam.xfam.org/).

**Figure 4:**
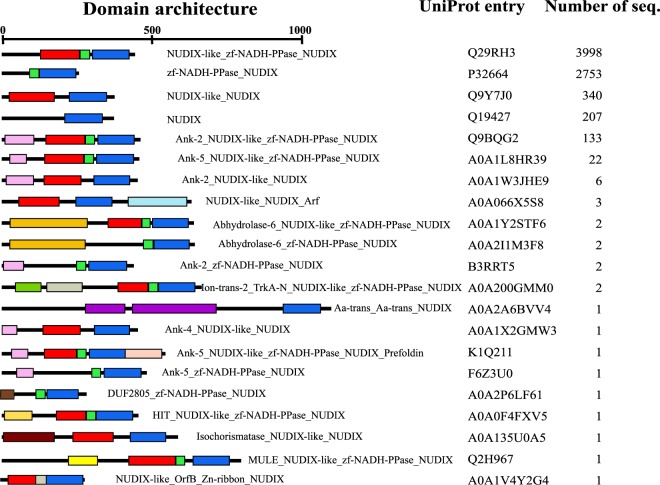
Domain architecture organization of the NADD subfamily. Domain architectures (21) are shown indicating the UniProtKB accession number of a representative sequence and the number of sequences with each architecture organization. The most important domains are coloured as in Fig. 3.

The ranking found in domain architecture in fungal NADD sequences is quite similar to that found in the 7479 NADDs used in this study, i.e. 53% for canonical NUDIX-like_zf-NADH-PPase_NUDIX, 37% for zf-NADH-PPa.5se_NUDIX, 4.5% for NUDIX-like_NUDIX, 2.8% for NUDIX and 1.8% for Ank2_NUDIX-like_zf-NADH-PPase_NUDIX (Fig. 4, Table 2). However, this last domain structure increases to 18.6% in eukaryotes. In the remaining domain architectures, which represent only 2%, are the typical NADDs domains combined with other different domains, such as a transmembrane amino acid transporter protein (Aa-trans), an alpha/beta hydrolase 6 family (Abhydrolase-6), a domain of unknown function (DUF2805), a histidine triad (HIT) present in nucleotide hydrolases that acts on the α-phosphate of ribonucleotides, an ion channel (Ion-trans-2), a domain that binds to NAD^+^ (TrkA-N), or a putative transposase DNA-binding domain (OrfB-Zn-ribbon) with four conserved cysteines, which could play a similar role to zf-NADH-PPase domain (Fig. 4). Finally, NADD architectures were also compared with that of the Nudix hydrolase family in PFAM (Table 2). Clearly, NUDIX domain alone was the most abundant (83.4%), and the sum of canonical NADD domain plus zf-NADH-PPase_NUDIX accounted for 3.9% of total Nudix hydrolases (Table 2). This percentage is similar to that obtained for the Prosite pattern described in this paper. The rest of the domain architectures are present at less than 0.1% in the NUDIX family, except for NUDIX-like_NUDIX (0.6%) (Table 2).

### MISTIC analysis reveals new important hydrophobic amino acids in fungal NADD sequences

**Figure 5:**
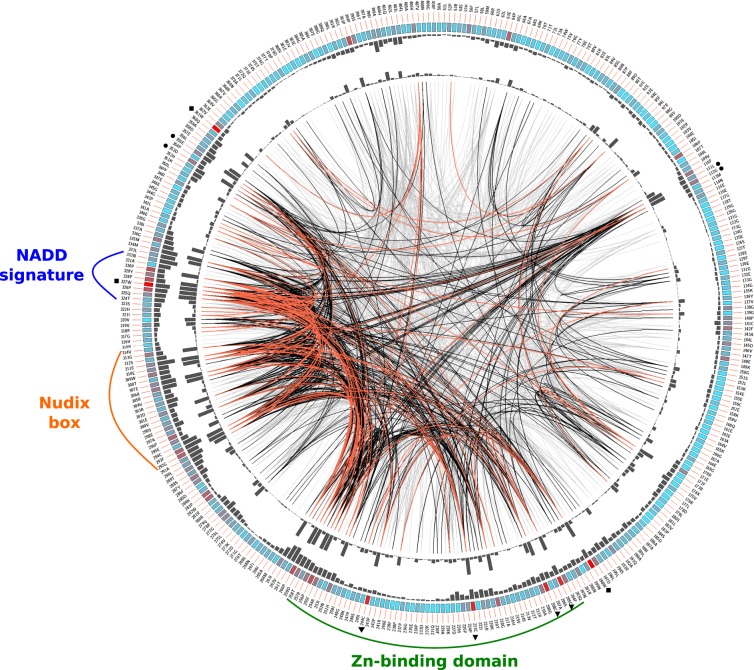
Circos representation of the fungal NADDs.The outer ring shows the amino acid code corresponding to TmNADD (UniProtKB accession number, D5GP45). Coloured rectangular boxes of the second circle indicate the KL (Kullback- Leibler) conservation score (from red to cyan, red: highest; cyan: lowest). The third circle shows the cMI (cumulative Mutual Information score) scores as histograms, which measure the degree of shared mutual information of a given residue. The fourth circle shows the pMI (proximity Mutual Information score), which describes the networks of mutual information in the proximity of a residue. Lines in the centre of the circle connect pairs of positions with MI (Mutational Information) score >6.5. Red lines represent the top 5%, the black lines between 70 and 95%, and the grey lines account for the last 70%. Sequence distribution of Zn-binding domain, Nudix box and NADD signature are shown. The four cysteines from the zinc-binding domain are marked with inverted triangles (▼). Residues with high cMI are marked with circles (●), whereas tryptophan conserved residues are marked with squares (■).

Mutual Information (MI) Server to Infer Coevolution (MISTIC) was used to predict positional correlations in the multiple sequence alignment (MSA) corresponding to NADDs sequences in order to guide the identification of structurally or functionally important positions in the fungal NADD family (Fig. 5)^30^. *T. melanosporum* NADD sequence (TmNADD, UniProtKB accession number: D5GP45) and its corresponding modelled structure were set as references. Circos representation shows that the information is basically concentrated in three main regions of the protein: residues 203–258 (Zn-binding domain), 292–314 (Nudix box) and 324–332 (NADD signature) (see outer histogram pMI and inner MI connection lines) (Fig. 5). However, previously undescribed regions can be found in the NTD (NUDIX-like domain), such as 108–114 and 172–202, with individual residues (hubs) with high cumulative Mutual Information (cMI) values (i.e., a large number of MI connections), such as L111, G112 and W198 (Fig. 5, solid circles). Similarly, another region (349–363) is found in the Nudix domain, with high cMI values in D353 and E355 (Fig. 5, solid circles). As regards conserved positions (Fig. 5, coloured square boxes of the second circle), the four cysteines in the zinc-binding domain are clearly highlighted (C205, C208, C223 and C244) in red (Fig. 5, triangles), together with three tryptophan residues (W198, W327 and W361) (Fig. 5, squares), pointing to functionally important residues in NADDs that remain to be studied (Fig. 5).

### Phylogenetic analysis of Pezizomycetes NADH pyrophosphatases agrees with their described molecular evolution

**Figure 6:**
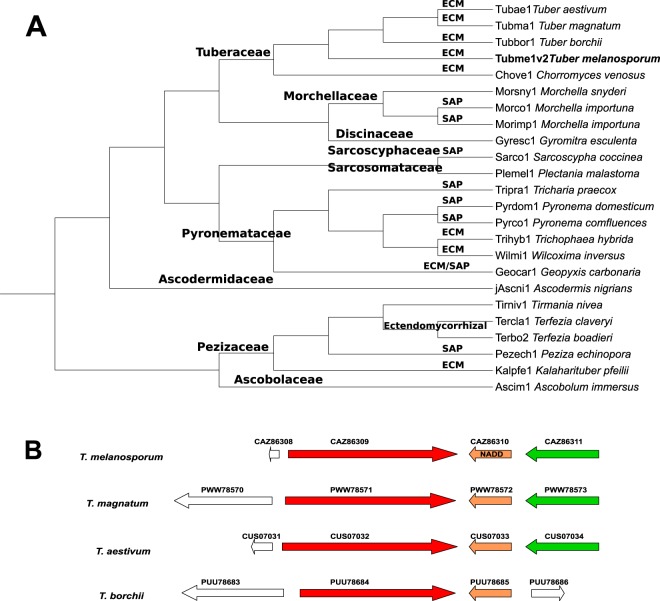
NADD distribution in Pezizomycetes. (**A**) Phylogenetic analysis of Pezizomycetes NADDs. The Neighbour-Joining (NJ) tree with 1000 replicates was constructed using MAFFT server. Saprotrophic (SAP) or ectomycorrizal (ECM) origin are indicated. Pezizalian sequences are summarized in Supplementary Table S2. (**B**) Comparative genome map of *T. melanosporum* gene CAZ86310 and those corresponding to *T. magnatum*, *T. aestivum* and *T. borchii*. Gene codifying the NADD proteins are marked in orange and the terpene cyclase/mutase family member gene in green.

In order to explore similar sequences in other Pezizomycetes, the fungal genomes described in the MycoCosm portal were also scanned (https://genome.jgi.doe.gov/mycocosm/home). A total of twenty-four sequences were found, including the five previously used from UniProtKB (Supplementary Table S2). The new phylogenetic NADD tree obtained showed two main clades (Fig. 6A). The first corresponds to the basal clade formed by two main sub-clades, those of Ascobolaceae (1) and Pezizaceae (5) families, respectively, in the last of which, most desert truffles (*Tirmania nivea*, *Terfezia claveryi* Chatin, *Te. boudieri* Chatin and *Kalaharituber pfeilii*) are found together with the saprophytic cup fungus *Peziza echinospora*. The second clade shows six sub-clades each corresponding to members of the Ascodesmidaceae (1), Pyronemataceae (6), Sarcosomataceae (1), Sarcoscyphaceae (1), Discinaceae (1), Morchellaceae (3) and Tuberaceae (5) families. The clades obtained with NADD proteins was in accordance with the phylogram generated from maximum likelihood analysis of combined LSU, SSU, ITS, TEF and RPB2 sequence data from 40 pezizalian species^25^. The latter study also showed Ascobolaceae (1) and Pezizaceae in basal clade 1, Ascodesmidaceae and Pyronemataceae in clade 6, Sarcosomataceae and Sarcoscyphaceae in clade 5, and finally Discinaceae, Morchellaceae and Tuberaceae in clade 4^25^. The above concordance with NADD distribution found for taxa of Pezizomycetes also agreed with the molecular phylogeny obtained using 2,093 concatenated conserved single-copy protein-coding genes from the eight Pezizomycetes^27^, in which NADD seems to be present in the most recent common ancestor (MRCA) of Pezizomycetes 470 ± 67 million years ago (Ma), starting from Ascobolaceae, and continuing present until Tuberaceae began diverging around 140 ± 10 Ma in the Early Cretaceous^27,31^. Interestingly, this MRCA seems to have the canonical NADD domain architecture (i.e. NUDIX-like_zf-NADH-PPase_NUDIX), since all the NADDs used in Fig. 6A have this pattern. Of note, in the NADD tree, *Choiromyces venosus* NADD forms a distinct branch from *Tuber* spp. within Tuberaceae, and *T. melanosporum* from the rest of *Tuber* spp., as previously described in the molecular phylogeny^27^.

Genome context analysis of the above selected sequences was also carried out, but not all genomes are annotated in Ensembl Fungi (http://fungi.ensembl.org/index.html). However, this is not the case for the above used *Tuber* spp., whose genome organization reveals a common pattern in NADD flanking genes (Fig. 6B). Thus, the NADD gene is preceded in all cases by a gene whose product is an uncharacterized protein of similar length (1517–1710 amino acids) (Fig. 6B, red), and followed by another gene whose product is a terpene cyclase/mutase family member of 719 amino acids length (Fig. 6B, green), except in the case of *T. borchii*, whose gene product is an uncharacterized protein of 323 amino acids. This difference in the genomic structure could also be related to the fact that *T. borchii* forms a sister phylogenetic group with respect to other *Tuber spp*^31^.

### TmNADD is a highly active NAD^+^ pyrophosphatase

After sequence-based bioinformatics analysis, the GSTUM_00011698001 gene (aka CAZ86310 in Ensembl Fungi) (Fig. 6B) from *T. melanosporum* (strain Mel28) (Perigord black truffle) was cloned into pET28a, transformed into *E. coli* Rosetta 2 and purified in two-steps, which combines an initial Ni^2+^ affinity chromatography step with a size exclusion in Superdex 200. The enzyme obtained was electrophoretically pure (Supplementary Fig. S2) and showed activity towards NAD^+^ in the presence of both Mn^2+^ or Mg^2+^, but with clear differences depending on the concentrations used (Supplementary Fig. S3), as previously described for EcNADD^11^. Manganese ion was clearly a better divalent metal ion than Mg^2+^, since lower concentrations are needed to reach maximal activity (0.5 mM *vs* 10 mM). In addition, Mn^2+^ gave rise to a 1.7-fold increase in activity at optimal conditions when compared with Mg^2+^ (Supplementary Fig. S3). However, at higher concentrations (>0.5 mM), Mn^2+^ showed an inhibitory effect (Supplementary Fig. S3). This preference for Mn^2+^ has also been described in human NUDT12 and mouse NUDT13, where it showed a 3-fold increase in activity compared to Mg^2+^^12,13^. That preference for Mn^2+^ seems to be a general characteristic in NADH pyrophosphatases, since it was also described for the *E. coli* and *S. cerevisiae* Nudix hydrolases^11,32^.

**Figure 7:**
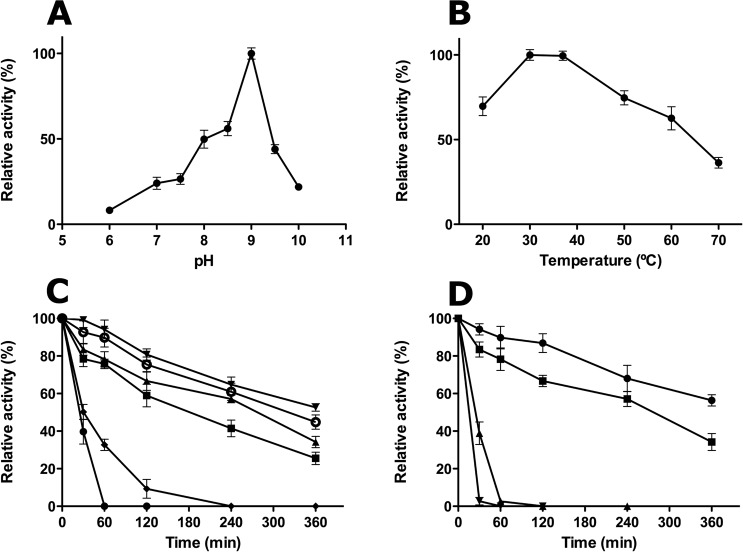
Effect of pH and temperature on TmNADD activity. (**A**) Effect of pH. Assay conditions were 0.5 mM MnCl_2_, 5 mM NAD^+^ and 0.11 μM of TmNADD in different 50 mM buffers at 37 °C. The activity was measured by HPLC for 15 min, as described in Materials and Methods. The buffers used were sodium phosphate pH 6.0–7.5, Tris-HCl pH 8.0–8.5, and glycine pH 9.0–10.0. (**B**) Effect of temperature. Assay conditions were the same as above but at pH 8.0 and different temperatures (20–70 °C). (**C**) Effect of pH on enzyme stability. TmNADD was incubated at 37 °C for different periods of time (0–360 min) at different pHs, and the activity was measured in the above standard reaction conditions. The buffers used (50 mM) were sodium phosphate pH 6.0 (●), pH 7.0 (■), Tris-HCl pH 8.0 (▲), pH 9.0 (○), and glycine pH 9.0 (▼), pH 10.0 (♦). (**D**) Effect of temperature on enzyme stability. The enzyme was incubated at pH 8.0 under the above standard conditions for different periods of time (0–360 min) at different temperatures [20 °C (●), 37 °C (■), 50 °C (▲) and 60 °C (▼)], and the activity was measured in the above standard reaction conditions. Data are the average of triplicate measurements.

The influence of the pH in the reaction catalysed by TmNADD was studied from pH 6.0 to pH 10.0, a clear optimum pH being observed at pH 9.0 (Fig. 7A), with a sharp decrease in activity above and below this value, especially at pH 9.5–10.0. This basic optimum pH was also found in other NADDs, such as hNUDT12 (pH 8.0–9.0), mouse NUDT13 (pH 8.2), EcNudC (pH 8.5) and CeNADD (pH 8.5)^11–14^, data which are consistent with the general alkaline nature of most Nudix hydrolases described up to date^2,5^. In addition, at pH 9.0, TmNADD was also more stable than at any other pH values (Fig. 7C), maintaining above 50% of its activity for 6 hours in glycine buffer (Fig. 7C, inverted triangles), whereas in Tris-HCl at the same pH it was about 8% less stable. This stability decreased gradually from pH 8.0 to pH 7.0, and was completely abolished in 1 hour at pH 6.0 (Fig. 7C, diamonds). However, at pH 10.0, the enzyme maintained 32% of its activity for the same time (Fig. 7C, closed circles). Such pH stability results cannot be compared, since no previous NADD stability studies have been carried out. For comparative purposes, the rest of the biochemical characterization was carried out at pH 8.0, as previously described^11–14^.

The temperature also affected TmNADD activity, with an optimal temperature between 30 °C and 37 °C at pH 8.0, and a steady decrease in activity above 40 °C, although maintaining 36% maximal activity at 70 °C (Fig. 7B). This optimal temperature was similar to that described for *Mycobacterium bovis* BCG NADD (MbNADD), which has an optimal temperature of 40 °C^19^. TmNADD displayed a half-life of about five hours at 37 °C, whereas at 20 °C it increased up to six hours (Fig. 7D). However, at temperatures above 37 °C, the enzyme half-life rapidly decreased to less than 60 minutes (Fig. 7D), only 2% remaining at 60 °C after 30 minutes.

As regards the substrate specificity, the enzyme showed a Michaelis-Menten kinetic towards both substrates (NADH and NAD^+^), but with a clear preference for NADH. Thus, the obtained K_M_ for NADH was 0.12 ± 0.01 mM, with a *k*_cat_ of 10.7 ± 1.8 s^−1^ and a *k*_cat_/K_M_ of 89.2 ± 19.8 mM^−1^ s^−1^, which is almost 10-fold higher than the catalytic efficiency shown towards NAD^+^ (*k*_cat_/K_M_ of 9.3 ± 1.6 mM^−1^ s^−1^) (Table 3). When comparing individually, the activity towards NAD^+^ and NADH, TmNADD showed the second best catalytic efficiency described to date towards both NAD^+^ and NADH, after human NUDT12 (Table 3). As regards NADH, it expressed higher catalytic efficiency than ScNADD and CeNADD (16- and 28-fold, respectively)^14^, but 11-fold lower than hNUDT12^13^. However, when NAD^+^ was considered, these differences increases up to 93-fold compared to ScNADD and CeNADD^14^, and decreased to 6-fold with respect to hNUDT12^13^. These results clearly indicate the preference of the above NADDs for NADH over NAD^+^^13,14^. However, no data are available in the literature about the reaction time needed to attain the full conversion of NAD^+^ into NMN and AMP. When TmNADD was assayed with 5 mM NAD^+^, complete conversion was obtained after 16 h (Supplementary Fig. S4).

**Table 3:** Kinetic parameters of TmNADD and other described NADH diphosphatases towards NAD^+^ and NADH.

Enzyme	Substrate	K_M_ (mM)	*k*_cat_ (s^−1^)	*k*_cat_/K_M_ (mM^−1^ s^−1^)	NAD^+^/NADH catalytic efficiency ratio
TmNADD	NAD^+^	0.31 ± 0.05	2.9 ± 0.2	9.3 ± 1.6	0.10
	NADH	0.12 ± 0.01	10.7 ± 1.8	89.2 ± 19.8	
ScNADD	NAD^+^	6.6	0.69	0.1	0.02
	NADH	1.6	8.9	5.56	
CeNADD	NAD^+^	6.6	0.63	0.1	0.03
	NADH	1.4	4.3	3.1	
hNUDT12	NAD^+^	0.19	10.5	55.2	0.06
	NADH	0.011	11.0	1000	

Kinetic parameters with the corresponding standard deviation were obtained as described in Materials and Methods section.

## Discussion

Nudix hydrolases are a vast and diverse family of proteins involved in the regulation of cellular responses and homeostasis, which are widely distributed in all kingdoms of life^9^. The Nudix box acts as an essential catalytic site of these enzymes, in which the glutamine residues (in bold) found in its sequence (GX_5_EX_7_R**E**UX**EE**XGU) bind indispensable divalent cations, such as Mg^2+^ or Mn^2+^^10^. Their substrate specificity is associated with additional sequences to this box, giving rise to the several subfamilies^3^. Among them, NADH pyrophosphatases or diphosphatases have been poorly studied, especially in enzymes of fungal origin. Taking advantage of the large number of new sequences emanating from genomic sequencing projects and their translation into the corresponding public protein databases, this work provides a complete *in silico* analysis of this subfamily, finding that NADDs represent 3.2% of the Nudix proteins in the UniProtKB database. Bacterial sequences are the most abundant, followed by eukaryotic, archaeal and metagenomic, without any viral representative. Curiously, fungal NADDs, distributed among different subphyla, account for 6.4% of NADDs and 68% of eukaryotic sequences, the most relevant being Pezizomycotina and Saccharomycotina (Fig. 2, Table 1). This abundance in Fungi is almost double those of the eukaryotic sequences of entire Nudix and Peptidase S8 families, as described in the Results section (Table 1). In addition, when these NADDs are compared within CATH Superfamily 3.90.79.10 (Supplementary Fig. S1D), which includes Nudix hydrolases (http://www.cathdb.info/version/v4_2_0/superfamily/3.90.79.10), fungal NADDs are also the most representative within structural cluster 3.90.79.10/3 (SC:3), where the nucleoside triphosphatases NudI, the ADP-ribose pyrophosphatases, the mitochondrial putative pre-mRNA cleavage factors, and the bifunctional NMN adenylyltransferases/Nudix hydrolases are also found. In fact, only one sequence from *Nosema bombycis* (strain CQ1 / CVCC 102059) (R0KWY1) was found in the nucleoside triphosphatase nudI sub-family when CATH sequences were retrieved from UniProt database.

NADDs are also found in Pezizomycetes basal groups, and in particular, in five economically important ectomycorrhizal truffle species, including the aromatic Périgord black truffle (*T. melanosporum*). The sequences of these truffle NADDs show the canonical domain architecture (i.e. NUDIX-like_zf-NADH-PPase_NUDIX) (Fig. 3), which is also the most common in both the fungal (54%) (Table 2) and in the 7479 NADDs (53%) sequences retrieved from UniProtKB (Fig. 4). This canonical architecture was also found in all the Pezizomycetes NADDs obtained from the MycoCosm portal, whose phylogenetic tree corroborates well with both the phylogram generated from the maximum likelihood analysis of combined LSU, SSU, ITS, TEF and RPB2 sequence data from 40 pezizalian species^25^ and the Pezizomycetes taxa distribution found in its molecular phylogeny obtained using 2,093 concatenated conserved single-copy protein-coding genes from the eight Pezizomycetes^27^. This suggests that NADD seems to be present in the most recent common ancestor (MRCA) of Pezizomycetes, and has been maintained as an essential protein from Ascobolaceae to Tuberaceae^27,31^.

The bioinformatic study also showed for the first time a total of twenty-one different architectures for the NADDs used in this work (Fig. 4), whereas only eleven were found in fungal sequences (Table 2). The human peroxisomal NUDT12 domain architecture (Ank2_NUDIX-like_zf-NADH-PPase_NUDIX) (Fig. 3, Table 2)^9^ accounts for 18.6% of the NADD domain architectures in eukaryotes, but only 0.42% in Fungi, in particular in the soil fungi *Bifiguratus adelaidae* (A0A261XXG3) and *Syncephalastrum racemosum* (A0A1X2H7S1). The latter fungus has occasionally been described as being the causative agent in toenail onychomycosis^33^. In addition, while the Ank-2 motif is found in 225,525 sequences with about 12,570 different Pfam domain architectures (http://pfam.xfam.org/family/Ank_2#tabview=tab0), the Ank-2_NUDIX-like_zf-NADH-PPase_NUDIX motif is only found in 88 sequences (http://pfam.xfam.org/family/zf-NADH-PPase#tabview=tab1) plus the two sequences found in this paper. This represents only 0.7% of the architectures where the Ank-2 motif is found. Its possible role associated with NADD is still unknown, although this Ank-2 domain is one of the most common protein-protein interaction platforms in nature. However, the presence of ankyrin domains (Ank-2, Ank-4 and Ank-5) in NADDs is relatively frequent both in fungal and eukaryotes, but totally absent in bacteria and archaea.

TmNADD is the first ectomycorrhizal Nudix NADH diphosphatase to be cloned and kinetically characterized, similar catalytic properties to those described previously for others NADDs being identified, but with some interesting particular features. As regards divalent cations, TmNADD had a preference for Mn^2+^ rather than Mg^2+^, with an optimal concentration (500 µM) higher than that described for hNUDT12 (50 µM) but similar to that of *S. cerevisiae* (300 µM)^13,32^. However, the optimal Mg^2+^ concentration was in the mM range (5–10 mM), similar to that described for *E. coli*, *S. cerevisiae* and *C. elegans*, but higher than that of hNUDT12 (0.4–2 mM)^11,13,14^. Its alkaline optimal pH was also a common feature of other NADDs, maintaining more than 50% activity for 6 hours in glycine buffer pH 9.0 at its optimum temperature (30–37 °C). Kinetically, TmNADD is 10 times more active towards NADH than NAD^+^. This ratio is lower than those of *E. coli* (120), *S. cerevisiae* (60), *C. elegans* (30) and even lower than that of hNUDT12 (20)^11,13,14^, meaning that the NAD^+^/NADH catalytic efficiency ratio is twice as high in TmNADD as in hNUDT12, making TmNADD the enzyme with the highest NAD^+^/NADH ratio ever described (Table 3). This observation, together with the fact that NAD^+^ is fully converted to NMN (Supplementary Fig. S4), and its easy expression and purification compared to hNUDT12, makes TmNADD a promising biocatalyst for the production of NMN from relatively cheap NAD^+^. NMN is a well-known NAD^+^-booster, capable of increasing the intracellular level of NAD^+^ after administration, increasing both life span and health in a number of animal models, and which is now being trialled in humans^34,35^.

## Materials and Methods

### Protein expression and purification

*Tuber melanosporum* Mel28 GSTUM_00011698001 gene, which was used for the cloning of the NADH pyrophosphatase/diphosphatase (TmNADD, UniprotKB accession number; D5GP45), was purchased from Genscript (NJ, USA) and inserted into pET28a vector. The TmNADD-pET28a construct was transformed into *Escherichia coli* Rosetta2(DE3) and induced using 0.25 mM isopropyl-β-D-thiogalactoside (IPTG) for 16 h at 20 °C with constant shaking. The culture was centrifuged and the pellet resuspended in lysis buffer (50 mM Tris-HCl pH 7.5, 150 mM NaCl). Cells were disrupted using a Bead Beater homogenizer (Biospec). After ultracentrifugation (40000 *g*, 40 min), the supernatant was loaded at 4 °C onto a HiPrep IMAC 16/10 FF column (GE Lifesciences, Spain) coupled to a FPLC chromatography system (ÄKTA Prime Plus, GE Lifesciences). The enzyme containing fractions were pooled, desalted and loaded into a Superdex 200 HiLoad 16/600 column (GE Lifesciences), obtaining an electrophoretically pure enzyme. TmNADD was stored at −20 °C with 10% glycerol.

### Characterization of the purified enzyme

Reactions towards NAD^+^ were carried out under standard NADD activity assay that have been described previously^11^. Reactions at 37 °C were prepared in 50 mM Tris-HCl pH 8.0 in the presence of 0.5 mM MnCl_2_, 5 mM NAD^+^ and 0.11 μM of TmNADD, unless otherwise stated. At regular intervals during the 15 min that the assay lasted, aliquots were taken, stopped at pH 3.0 with TFA (1% final concentration), kept on ice for 10 min and centrifuged for 10 min at 12000 *g*. NAD^+^ conversion into NMN and AMP was followed by HPLC in a reverse phase C18 column (Gemini C18 250 × 4.6 mm, Phenomenex) and a method involving buffer A (10 mM tetrabutylammonium bromide, 10 mM potassium phosphate pH 7.0 and 0.25% methanol) running at 1 mL min^−1^ in a gradient from 0 to 100% of buffer B (2.8 mM tetrabutylammonium bromide, 100 mM potassium phosphate pH 5.5 and 30% methanol) for 21 minutes. In these conditions, the reaction products NMN and AMP had a retention time of 5.1 and 13.1 minutes, respectively, and NAD^+^ had a retention time of 10.7 minutes. NAD^+^ diphosphatase activity was determined following the increase in the area of the AMP peak. Reactions towards NADH (5 mM) were carried out under the same conditions for 5 min, but using 0.02 μM of TmNADD. The reactions were stopped at regular intervals by the addition of 100 mM EDTA at pH 8.0^6^. Under these conditions, the reaction products NMNH and AMP had retention times of 15.2 and 13.1 minutes, respectively, while that of NADH was 19.1 minutes. NADH diphosphatase activity was also determined following the increase in the area of the AMP peak.

The optimal pH of TmNADD was carried out by performing the standard activity assay in various buffers with different pH values: 6.0–10.0. The buffers used were as follows: sodium phosphate buffer (pH 6.0–7.5), Tris-HCl buffer (pH 8.0–9,0), and glycine-NaOH buffer (pH 9.0–10.0). All buffers were at a concentration of 50 mM. Next, optimum temperature for TmNADD was determined using the standard NADD activity assay described above at different temperatures ranging from 20 to 60 °C.

The effect of pH and temperature on enzyme stability were determined by incubating TmNADD at 37 °C at different pHs, or at pH 8.0 at different temperatures for increasing periods of time (0–360 min). The residual activity after the incubation period was measured in the standard reaction conditions.

The substrate specificity of TmNADD was determined at 37 °C and in 50 mM Tris-HCl pH 8.0 using various substrates. The Michaelis-Menten constant (K_M_) and maximal velocity (*V*_*max*_) were estimated using plots of initial rates vs. NADH or NAD^+^ concentrations, respectively. The reported values represent means ± SE of the fits of the curves to the Michaelis-Menten equation by non-linear regression based on triplicate experiments. The catalytic constant (*k*_cat_) and the catalytic efficiency (*k*_cat_ /K_M_) were deduced from the obtained K_M_ and *V*_*max*_ values.

### *In silico* analysis

Protein sequences were obtained from the UniProtKB database (https://www.uniprot.org/), using the Prosite pattern PS51462 (NUDIX) as a query. Incomplete sequences and duplicate were removed, rendering the sequences used in the study. The NADD-like sequences were identified using the ScanProsite application (https://prosite.expasy.org/scanprosite/) and the pattern designed in this work. Sequences were aligned with the default parameters using MAFFT server (https://mafft.cbrc.jp/alignment/server/) and displayed using ESPript 3.0^36^. Subsequently, the tree was built on the same web server using the Neighbour-Joining (NJ) method, JTT substitution model, heterogeneity between ignored sites (α = ∞) and a bootstrap of 1000 replicates to increase the reliability of the tree obtained. Mutation correlation analysis was carried out with the retrieved fungal NADD sequences using MISTIC (Mutual Information Server to Infer Coevolution) web server (http://mistic.leloir.org.ar/)^30^. The domain architectures of retrieved sequences were obtained from the Pfam (https://pfam.xfam.org/) and visualized with iTOL (https://itol.embl.de/).

## Supplementary information

Supplementary Information
