# Supplementary material for: The first comprehensive phylogenetic and biochemical analysis of NADH diphosphatases reveals that the enzyme from Tuber melanosporum is highly active towards NAD+
Source: Sci Rep. 2019 Nov 14;9:16753. doi: 10.1038/s41598-019-53138-w (PMC6856373; doi:10.1038/s41598-019-53138-w)
Supplement: Supplementary file 1 — Supplementary Information [file 41598_2019_53138_MOESM1_ESM.pdf]

# Supplementary Information

## The first comprehensive phylogenetic and biochemical analysis of NADH diphosphatases reveals that the enzyme from *Tuber melanosporum* is highly active towards $NAD^+$

**Authors:**

Antonio Ginés García-Saura[1,4], Rubén Zapata-Pérez[1,2], Ana Belén Martínez-Moñino[1,4], José Francisco Hidalgo[1], Asunción Morte[3], Manuela Pérez-Gilabert[1,4] & Álvaro Sánchez-Ferrer[1,4,*]

**Affiliations:**

[1]Department of Biochemistry and Molecular Biology-A, Faculty of Biology, Regional Campus of International Excellence "Campus Mare Nostrum", University of Murcia, Campus Espinardo, 30100 Murcia, Spain.

[2]Lab. Genetic Metabolic Diseases F0-211, Academic Medical Center (AMC), 1105 AZ Amsterdam, The Netherlands

[3]Department of Plant Biology, Faculty of Biology, University of Murcia, Campus Espinardo, 30100 Murcia, Spain.

[4]Murcia Biomedical Research Institute (IMIB-Arrixaca), 30120 Murcia, Spain.

*Corresponding author A.S-F.; alvaro@um.es

| UniProtKB code | Specie | Order | UniProtKB code | Specie | Order |
|---|---|---|---|---|---|
| A0A166AFX6 | *Exidia glandulosa* HHB12029 | Auriculariales | C4JM48 | *Uncinocarpus reesii* (strain UAMH 1704) | Onygenales |
| A0A1Y1X6L1 | *Basidiobolus meristosporus* CBS 931.73 | Basidiobolales | F0UPP7 | *Ajellomyces capsulatus* (strain H88) | Onygenales |
| A0A0C9ZYI5 | *Pisolithus microcarpus* 441 | Boletales | A0A022W160 | *Trichophyton rubrum* CBS 288.86 | Onygenales |
| K2RVI2 | *Macrophomina phaseolina* (strain MS6) | Botryosphaeriales | E4URS2 | *Arthroderma gypseum* (strain ATCC MYA-4604) | Onygenales |
| A0A0G2EIQ8 | *Diplodia seriata* | Botryosphaeriales | A0A2B7XUG2 | *Polytolypa hystricis* UAMH7299 | Onygenales |
| N1PQ83 | *Dothistroma septosporum* (strain NZE10) | Capnodiales | A0A0G2I7W8 | *Emmonsia crescens* UAMH 3008 | Onygenales |
| A0A1V8TRB8 | *Rachicladosporium antarcticum* | Capnodiales | A0A1J9QI37 | *Blastomyces percursus* | Onygenales |
| A0A2D3UYQ6 | *Ramularia collo-cygni* | Capnodiales | A0A1J9QMI8 | *Emergomyces pasteurianus* Ep9510 | Onygenales |
| A0A2H1GT25 | *Zymoseptoria tritici* ST99CH_1E4 | Capnodiales | C5FI71 | *Arthroderma otae* (strain ATCC MYA-4605) | Onygenales |
| A0A2G5HH78 | *Cercospora beticola* | Capnodiales | A0A167WT13 | *Ascosphaera apis* ARSEF 7405 | Onygenales |
| A0A139IEP0 | *Pseudocercospora musae* | Capnodiales | A0A0J6I7W4 | *Coccidioides posadasii* RMSCC 3488 | Onygenales |
| M2LIA8 | *Baudoinia panamericana* (strain UAMH 10762) | Capnodiales | A0A1D2JDH7 | *Paracoccidioides brasiliensis* | Onygenales |
| A0A139H9E7 | *Mycosphaerella eumusae* | Capnodiales | A0A2B7XTL0 | *Helicocarpus griseus* UAMH5409 | Onygenales |
| N1QII2 | *Sphaerulina musiva* (strain SO2202) | Capnodiales | F0XAG0 | *Grosmannia clavigera (*strain kw1407) | Ophiostomatales |
| A0A150USA1 | *Acidomyces richmondensis* BFW | Capnodiales | S3CR68 | *Ophiostoma piceae* (strain UAMH 11346) | Ophiostomatales |
| A0A0D2GND0 | *Fonsecaea pedrosoi* CBS 271.37 | Chaetothyriales | A0A0C2JDB7 | *Sporothrix brasiliensis* 5110 | Ophiostomatales |
| W2RLD3 | *Cyphellophora europaea* CBS 101466 | Chaetothyriales | G1X2G6 | *Arthrobotrys oligospora* (strain ATCC 24927) | Orbiliales |
| A0A0D2G4N6 | *Rhinocladiella mackenziei* CBS 650.93 | Chaetothyriales | S8A4N1 | *Dactylellina haptotyla* (strain CBS 200.50) | Orbiliales |
| W9YI54 | *Capronia epimyces* CBS 606.96 | Chaetothyriales | W7HPD1 | *Drechslerella stenobrocha 248* | Orbiliales |
| A0A072NWT6 | *Exophiala aquamarina* CBS 119918 | Chaetothyriales | U4LP02 | *Pyronema omphalodes* (strain CBS 100304) | Pezizales |
| A0A0D2FKR7 | *Phialophora americana* | Chaetothyriales | A0A2T6ZTD3 | *Tuber borchii* | Pezizales |
| A0A0D1ZP06 | *Cladophialophora immunda* | Chaetothyriales | D5GP45 | *Tuber melanosporum* (strain Mel28) | Pezizales |
| A0A1Y2CYD5 | *Rhizoclosmatium globosum* | Chytridiales | A0A292PI49 | *Tuber aestivum* | Pezizales |
| A0A1J7J2F8 | *Coniochaeta ligniaria* NRRL 30616 | Coniochaetales | A0A317SZB3 | *Tuber magnatum* | Pezizales |
| A0A0G2FVZ6 | *Diaporthe ampelina* | Diaporthales | A0A0L1HN44 | *Stemphylium lycopersici* | Pleosporales |
| A0A194VHB3 | *Valsa mali var. pyri* | Diaporthales | A0A177DS68 | *Alternaria alternata* | Pleosporales |
| A0A2T3AF81 | *Coniella lustricola* | Diaporthales | A0A2W1FX52 | *Pyrenophora tritici-repentis* | Pleosporales |
| A0A261XXG3 | *Bifiguratus adelaidae* | Endogonales | M2RL04 | *Cochliobolus sativus* (strain ND90Pr) | Pleosporales |
| A0A137PCQ8 | *Conidiobolus coronatus* (strain ATCC 28846) | Entomophthorales | R0K7A1 | *Setosphaeria turcica* (strain 28A) | Pleosporales |
| B8MDE6 | *Talaromyces stipitatus* (strain ATCC 10500) | Eurotiales | A0A2T2PB47 | *Corynespora cassiicola Philippines* | Pleosporales |
| A0A0F4YR44 | *Rasamsonia emersonii* CBS 393.64 | Eurotiales | A0A177C9U5 | *Paraphaeosphaeria sporulosa* | Pleosporales |
| V5FY54 | *Byssochlamys spectabilis* (strain No. 5 / NBRC 109023) | Eurotiales | A0A1Y2LSW0 | *Epicoccum nigrum* | Pleosporales |
| A0A0A2JRW0 | *Penicillium expansum* | Eurotiales | A0A178B4F9 | *Stagonospora sp.* SRC1lsM3a | Pleosporales |
| A0A1L9SMS2 | *Penicilliopsis zonata* CBS 506.65 | Eurotiales | Q0TYJ0 | *Phaeosphaeria nodorum* (strain SN15) | Pleosporales |
| G3XLL1 | *Aspergillus niger* (strain ATCC 1015) | Eurotiales | A0A178DST1 | *Pyrenochaeta sp.* DS3sAY3a | Pleosporales |
| A0A232LZZ7 | *Elaphomyces granulatus* | Eurotiales | E5A9C9 | *Leptosphaeria maculans* (strain JN3) | Pleosporales |
| A0A010RIZ9 | *Colletotrichum fioriniae* PJ7 | Glomerellales | A0A163KLM4 | *Didymella rabiei* | Pleosporales |
| A0A0G4LU46 | *Verticillium longisporum* | Glomerellales | A0A0L0VEI9 | *Puccinia striiformis f. sp. tritici* PST-78 | Pucciniales |
| A0A066X5S8 | *Colletotrichum sublineola* | Glomerellales | A0A0C7MXP8 | *Lachancea lanzarotensis* | Saccharomycetales |
| A0A135U0A5 | *Colletotrichum salicis* | Glomerellales | A0A1E3NYD0 | *Wickerhamomyces anomalus* NRRL Y-366-8 | Saccharomycetales |
| A0A2T9YYB4 | *Furculomyces boomerangus* | Harpellales | G0WFT4 | *Naumovozyma dairenensis* (strain ATCC 10597) | Saccharomycetales |
| A0A2T9XWV5 | *Smittium megazygosporum* | Harpellales | A0A1Q2ZXQ5 | *Zygosaccharomyces rouxii* | Saccharomycetales |
| N1S6E2 | *Fusarium oxysporum f. sp. cubense* (strain race 4) | Hypocreales | A0A1E4TPH1 | *Pachysolen tannophilus* NRRL Y-2460 | Saccharomycetales |
| A0A2A9PJ54 | *Ophiocordyceps unilateralis* | Hypocreales | H2B1T5 | *Kazachstania africana* (strain ATCC 22294) | Saccharomycetales |

| | | | | | |
|---|---|---|---|---|---|
| A0A151GWL8 | *Drechmeria coniospora* | Hypocreales | G8ZVX3 | *Torulaspora delbrueckii* (strain ATCC 10662) | Saccharomycetales |
| A0A0L0NE62 | *Tolypocladium ophioglossoides* CBS 100239 | Hypocreales | A0A1E4TLZ0 | *Tortispora caseinolytica* NRRL Y-17796 | Saccharomycetales |
| A0A0F9XB02 | *Trichoderma harzianum* | Hypocreales | A0A1E3QGH2 | *Lipomyces starkeyi* NRRL Y-11557 | Saccharomycetales |
| A0A0A1TB65 | *Torrubiella hemipterigena* | Hypocreales | E7NHN5 | *Saccharomyces cerevisiae* (strain FostersO) | Saccharomycetales |
| A0A0A1V141 | *Metarhizium robertsii* | Hypocreales | A0A1E4S719 | *Cyberlindnera jadinii* NRRL Y-1542 | Saccharomycetales |
| A0A179FQT9 | *Pochonia chlamydosporia* 170 | Hypocreales | B6K3A7 | *Schizosaccharomyces japonicus* (strain yFS275) | Schizosaccharomycetales |
| A0A0F7ZTB4 | *Hirsutella minnesotensis* 3608 | Hypocreales | G0S500 | *Chaetomium thermophilum* (strain DSM 1495) | Sordariales |
| A0A179HCG2 | *Purpureocillium lilacinum* | Hypocreales | G2QBS1 | *Myceliophthora thermophila* (strain ATCC 42464) | Sordariales |
| A0A086SXE9 | *Acremonium chrysogenum* (strain ATCC 11550) | Hypocreales | G2RAL4 | *Thielavia terrestris* (strain ATCC 38088) | Sordariales |
| A0A0P7BW75 | *Neonectria ditissima* | Hypocreales | A0A175VNB8 | *Madurella mycetomatis* | Sordariales |
| A0A0M8N229 | *Escovopsis weberi* | Hypocreales | F7VYD9 | *Sordaria macrospora* (strain ATCC MYA-333) | Sordariales |
| A0A084AGA7 | *Stachybotrys chartarum* (strain CBS 109288) | Hypocreales | B2AW63 | *Podospora anserina* (strain S / ATCC MYA-4624) | Sordariales |
| A0A166YHF9 | *Cordyceps brongniartii* RCEF 3172 | Hypocreales | A0A0B0EBA1 | *Neurospora crassa* | Sordariales |
| A0A0A2V9E6 | *Beauveria bassiana* D1-5 | Hypocreales | Q2H967 | *Chaetomium globosum* (strain ATCC 6205) | Sordariales |
| M1VY82 | *Claviceps purpurea* (strain 20.1) | Hypocreales | A0A061AE98 | *Rhodosporidium toruloides* | Sporidiobolales |
| A0A1Y2F8Q6 | *Leucosporidium creatinivorum* | Leucosporidiales | A0A0D6EGM5 | *Sporidiobolus salmonicolor* | Sporidiobolales |
| J3NL17 | *Gaeumannomyces graminis var. tritici* (strain R3-111a-1) | Magnaporthales | R8BIP2 | *Togninia minima* (strain UCR-PA7) | Togniniales |
| L7IHU1 | *Magnaporthe oryzae* (strain Y34) | Magnaporthales | A0A1A6A125 | *Kwoniella dejecticola* CBS 10117 | Tremellales |
| A0A0F4Z8M2 | *Thielaviopsis punctulata* | Microascales | A0A1E3ISW0 | *Tsuchiyaea wingfieldii* CBS 7118 | Tremellales |
| A0A0F8BXY9 | *Ceratocystis fimbriata f. sp. platani* | Microascales | A0A1Y1ULI6 | *Kockovaella imperatae* | Tremellales |
| A0A2N3NJP0 | *Lomentospora prolificans* | Microascales | A0A1Y2BLR2 | *Naematelia encephala* | Tremellales |
| A0A084G552 | *Scedosporium apiospermum* | Microascales | A0A226BE63 | *Cryptococcus neoformans var. grubii* Bt85 | Tremellales |
| G7E5P7 | *Mixia osmundae* (strain CBS 9802) | Mixiales | J4UCX7 | *Trichosporon asahii var. asahii* (strain ATCC 90039) | Trichosporonales |
| A0A139AV93 | *Gonapodya prolifera* JEL478 | Monoblepharidales | A0A0D1DMQ6 | *Ustilago maydis* (strain 521 / FGSC 9021) | Ustilaginales |
| A0A197JMS3 | *Mortierella elongata* AG-77 | Mortierellales | A0A0D1YL90 | *Verruconis gallopava* | Venturiales |
| A0A1Y2GRX2 | *Lobosporangium transversale* | Mortierellales | I4Y7Y0 | *Wallemia mellicola* (strain ATCC MYA-4683) | Wallemiales |
| A0A068RVP2 | *Lichtheimia corymbifera* JMRC:FSU:9682 | Mucorales | A0A136JAX7 | *Microdochium bolleyi* | Xylariales |
| A0A1X2H7S1 | *Syncephalastrum racemosum* | Mucorales | A0A1W2TET3 | *Rosellinia necatrix* | Xylariales |
| A0A1X2GMW3 | *Hesseltinella vesiculosa* | Mucorales | A0A1Y2WCP4 | *Hypoxylon sp.* CI-4A | Xylariales |
| A0A1C7NCR5 | *Choanephora cucurbitarum* | Mucorales | A0A1Y2XGD7 | *Daldinia sp.* EC12 | Xylariales |
| A0A167QH99 | *Phycomyces blakesleeanus* (strain ATCC 8743b) | Mucorales | W3X3L3 | *Pestalotiopsis fici* (strain W106-1) | Xylariales |
| A0A1Y1VG01 | *Piromyces finnis* | Neocallimastigales | A0A1Y2EDR7 | *Pseudomassariella vexata* | Xylariales |
| A0A1Y2FJ97 | *Neocallimastix californiae* | Neocallimastigales | M7TFR5 | *Eutypa lata* (strain UCR-EL1) | Xylariales |
| A0A1Y1X5F3 | *Anaeromyces robustus* | Neocallimastigales | A0A093XXH5 | *Pseudogymnoascus sp.* VKM F-3557 | |
| | | | A0A1V1TE82 | *fungal sp.* No.14919 | |

**Supplementary Table S1.** Fungal NAD pyrophosphatase sequences found in UniProtKB database. These sequences were used in the phylogenetic analysis of Fig. 3.

| Organism | MycoCosm ID | Protein ID |
|---|---|---|
| *Tuber aestivum var. urcinatum* | Tubae1 | 5814 |
| *Tuber magnatum* | Tubma1 | 350675 |
| *Tuber melanosporum* Mel28 | Tubme1v2 | 9591 |
| *Tuber borchii* Tbo3840 | Tubbor1 | 981933 |
| *Choiromyces venosus* 120613-1 | Chove1 | 1800574 |
| *Morchella importuna* SCYDJ1 | Morimp1 | 536297 |
| *Morchella importuna* CCBAS932 | Morco1 | 539244 |
| *Morchella snyderi* DOB2414 | Morsny1 | 530042 |
| *Gyromitra esculenta* CBS101906 | Gyresc1 | 556048 |
| *Sarcoscypha coccinea* ATCC 58028 | Sarco1 | 405854 |
| *Plectania melastoma* CBS 918.72 | Plemel1 | 434645 |
| *Tricharina praecox* DOB2270 | Tripra1 | 394161 |
| *Pyronema domesticum* DOB7353 | Pyrdom1 | 367252 |
| *Pyronema confluens* CBS100304 | Pyrco1 | 8892 |
| *Trichophaea hybrida* UTF0779 | Trihyb1 | 829633 |
| *Geopyxis carbonaria* DOB1671 | Geocar1 | 620284 |
| *Ascodesmis nigricans* CBS 389.68 | jAscni1 | 344341 |
| *Tirmania nivea* G3 | Tirniv1 | 52604 |
| *Terfezia claveryi* T7 | Tercla1 | 1096083 |
| *Terfezia boudieri* ATCC MYA-4762 | Terbo2 | 780713 |
| *Peziza echinospora* DOB1120 | Pezech1 | 601459 |
| *Kalaharituber pfeilii* F3 | Kalpfe1 | 722024 |
| *Ascobolus immersus* RN42 | Ascim1 | 321952 |
| *Wilcoxima inversus* | Wilmi1 | 627062 |

**Supplementary Table S2.** Pezizales NAD pyrophosphatase sequences found in UniProtKB and MycoCosm databases. These sequences were used in the phylogenetic analysis of Fig. 6A.

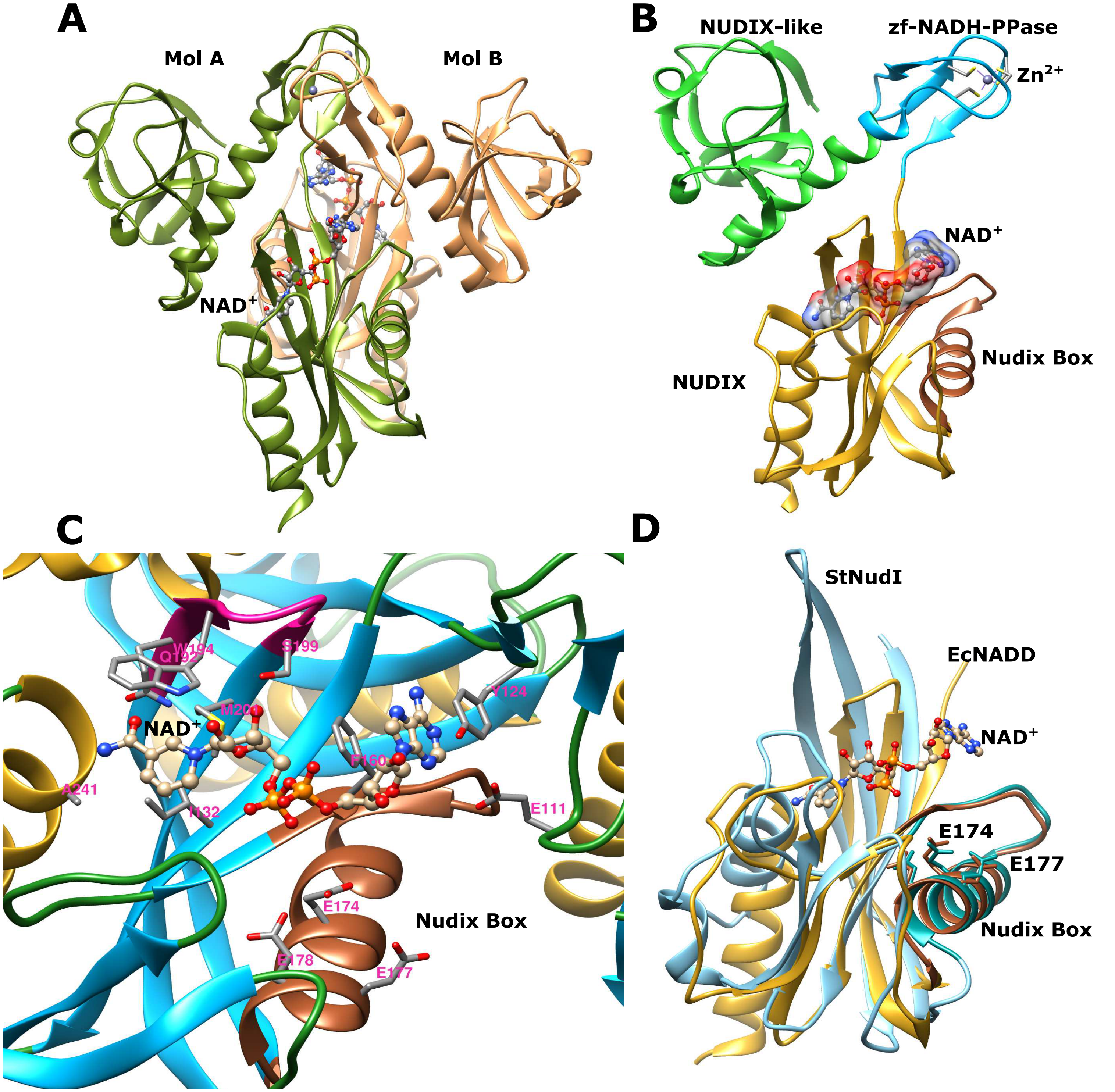


**Supplementary Figure S1.** Representation of the canonical NADD architecture. (**A**) Dimeric structure *E. coli* NADD (pdb:5ISY) with bound NAD$^+$. The two monomers (A and B) are shown in olive green and tan, respectively. The bound NAD$^+$molecules are shown in a CPK ball and stick representation. Zn$^{2+}$ is in purple (**B**) Detailed structural representation of a monomer of EcNADD-NAD$^+$complex. The NTD (aka NUDIX-like), zinc-binding motif (aka zf_NADH_PPase), and Nudix motif (aka NUDIX) are shown in green, cyan, and yellow, respectively. Nudix box is in brown. Zinc ion (purple) coordination with four cysteines is highlighted with purple lines. (**C**) The Prosite pattern and NAD$^+$ binding pocket. Residues involved in NAD$^+$binding are shown as sticks, Nudix box is in brown, whereas NADD signature (191-199) is in pink. (**D**) Structural alignment of the canonical Nudix hydrolase motif in two members of the CATH Superfamily 3.90.79.10 structural cluster 3 (SC:3). The structure of (EcNADD) (yellow) has been superimposed over the *Salmonella typhimurium* LT2 nucleoside triphosphatase NudI structure (cyan)(pdb: 3n77). Conserved glutamate (E) residues of Nudix box are shown in sticks (E174, E177 and E178 for EcNADD; E53, E56 and E57 for StNudI).

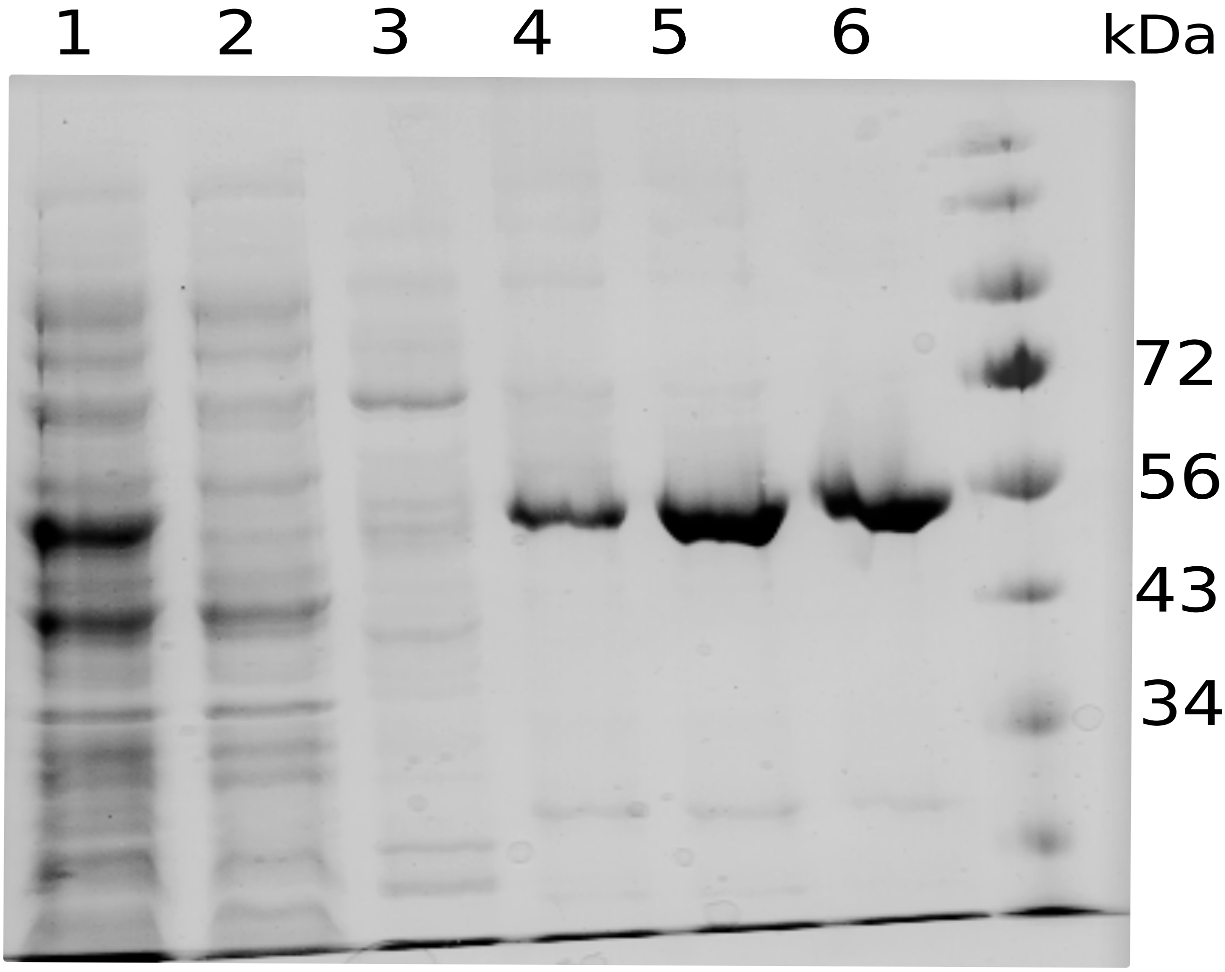


**Supplementary Figure S2**. TmNADD purification process. SDS-PAGE (10%) was used to follow the purification process. Lane 1: crude extract of induced TmNADD-pET28a-Rosetta 2 clone. Lane 2: crude extract of induced pET28a-Rosetta 2 clone. Lane 3: HisTrap flowthrough. Lane 4 and 5: HisTrap fractions. Lane 6: Superdex fraction. Lane 7: molecular weight standards (Fisher EZ-Run; BP3603-1).

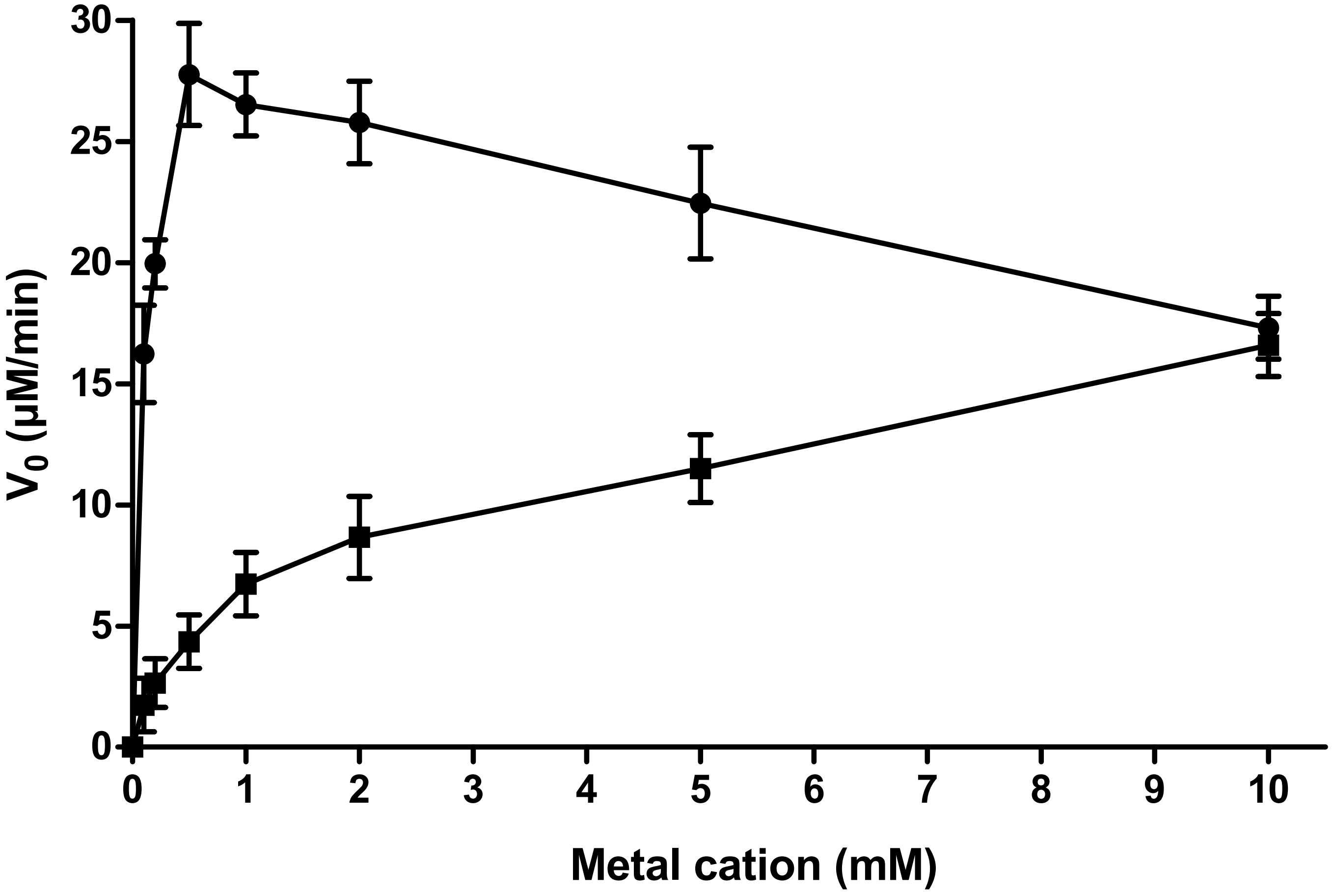


**Supplementary Figure S3**. Effect of divalent metal cations on TmNADD activity. Initial velocity *vs.* concentration of manganese ($Mn^{2+}$, ●) or magnesium ($Mg^{2+}$, ■) was plotted using $NAD^+$as a substrate.

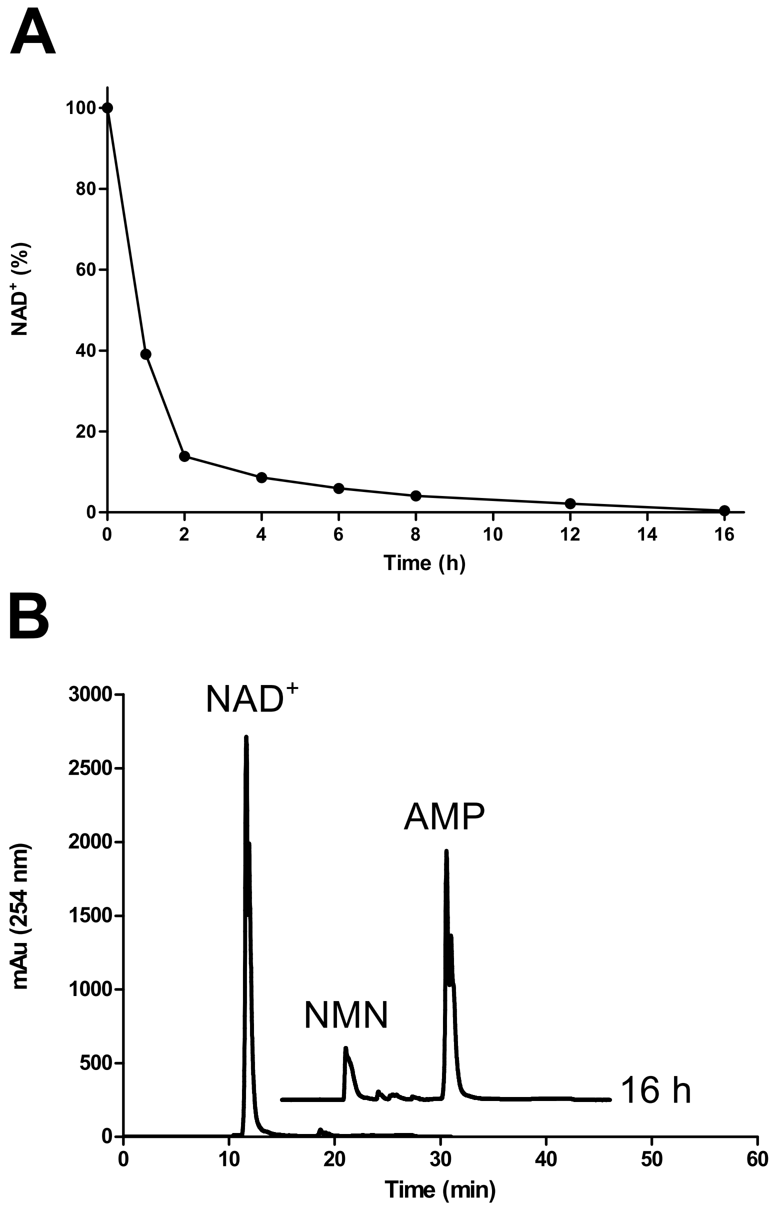


**Supplementary Figure S4.** Bioconversion of $NAD^+$by TmNADD.(**A**) Time course consumption of 5 mM $NAD^+$catalysed by TmNADD (100 μg) under the standard reaction conditions. (**B**) HPLC chromatograms at different reaction times (0-16 h).
